# Mask then classify: multi-instance segmentation for surgical instruments

**DOI:** 10.1007/s11548-021-02404-2

**Published:** 2021-06-18

**Authors:** Thomas Kurmann, Pablo Márquez-Neila, Max Allan, Sebastian Wolf, Raphael Sznitman

**Affiliations:** 1 ARTORG, University of Bern, Bern, Switzerland; 2 Intuitive Surgical Inc., Sunnyvale, USA; 3 Department of Ophthalmology, Bern University Hospital, Bern, Switzerland

**Keywords:** Instance segmentation, Surgical robotics, Deep learning

## Abstract

**Purpose:**

The detection and segmentation of surgical instruments has been a vital step for many applications in minimally invasive surgical robotics. Previously, the problem was tackled from a semantic segmentation perspective, yet these methods fail to provide good segmentation maps of instrument types and do not contain any information on the instance affiliation of each pixel. We propose to overcome this limitation by using a novel instance segmentation method which first masks instruments and then classifies them into their respective type.

**Methods:**

We introduce a novel method for instance segmentation where a pixel-wise mask of each instance is found prior to classification. An encoder–decoder network is used to extract instrument instances, which are then separately classified using the features of the previous stages. Furthermore, we present a method to incorporate instrument priors from surgical robots.

**Results:**

Experiments are performed on the robotic instrument segmentation dataset of the 2017 endoscopic vision challenge. We perform a fourfold cross-validation and show an improvement of over 18% to the previous state-of-the-art. Furthermore, we perform an ablation study which highlights the importance of certain design choices and observe an increase of 10% over semantic segmentation methods.

**Conclusions:**

We have presented a novel instance segmentation method for surgical instruments which outperforms previous semantic segmentation-based methods. Our method further provides a more informative output of instance level information, while retaining a precise segmentation mask. Finally, we have shown that robotic instrument priors can be used to further increase the performance.

**Supplementary Information:**

The online version supplementary material available at 10.1007/s11548-021-02404-2.

## Introduction

Minimally invasive surgical robotic systems such as the Intuitive Surgical da Vinci have seen widespread adoption for surgical procedures. Typically, these systems are operated in a teleoperative manner, where a surgeon controls instruments through visual feedback of an endoscope. The video feed of the endoscope allows the possibility to enhance, augment and even partially replace a human controller. Applications include pose estimation and tracking of surgical instruments [1, 3, 4, 11, 14] and virtual reality augmentation of the surgeons perception [2].

**Fig. 1:**
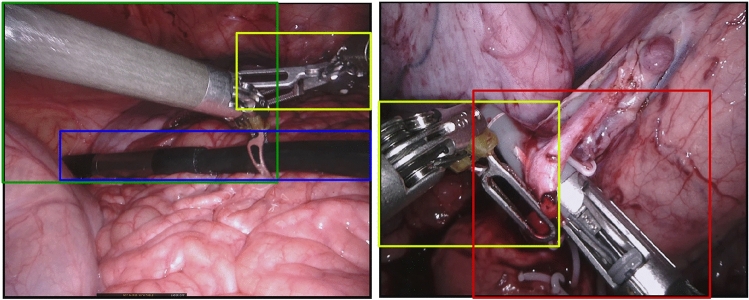
Example of bounding boxes which have large overlapping sections. Note how the green bounding box on the left image capture three different instruments inside its box

A central topic in these applications is the correct identification of surgical instruments, where the main focus so far has been the segmentation of the instruments [2, 7, 9, 13]. These methods have shown promising performance for binary segmentation, but have under-performed in *instrument type* segmentation tasks. These short-comings are mainly due to two reasons: (1) many surgical instruments share the same shaft and/or wrist and only differ in the clasper, and (2) instruments can occupy large portions of the image, requiring global context to be shared amongst pixels far away. These reasons lead to instrument instances being segmented incorrectly or containing multiple different labels. That is, even if these methods provided a perfect segmentation, the task of assigning an instance label would still be impractical. For one, disconnected regions of the same instrument, for example when an instrument is partially occluded, could not be assigned to the same instance. Furthermore, overlapping instruments of the same class would be considered a single instance. Only recently has the focus of the community shifted to the more complex task of instance segmentation [5, 8] where every instrument object is separated into an instance while simultaneously segmenting pixels belonging to it. Instance segmentation provides a natural way of separating object instances and their pixels, which is vital for applications such as instrument pose estimation and tracking. Pose estimation applications which make use of 3D models [1] require both the segmentation masks and instance type as input. Furthermore, these methods are agnostic to robotic and non-robotic surgeries where typically no prior information to instrument presence is available.

These methods rely on bounding box object detection methods adopted from methods designed for natural images. Yet, bounding boxes are not suitable for instruments that typically span larger spatial sizes with unequal length-width ratios.

Specifically, previous methods have focused on the problem from the view of semantic segmentation and only a few as instance segmentation [5, 8]. The problem at hand is more complex as it requires the proper separation of objects into their instances whilst segmenting their pixels. Many methods in the scope of instance segmentation have expanded methods from object detection with a subsequent segmentation part, a well-known example of this is Mask-RCNN [6]. These methods make heavy use of bounding boxes around the objects to latter perform segmentation. We argue that the concept of bounding boxes is ill-suited for the task at hand. For one, the instruments vary significantly in size and most importantly in angle. An instrument which is located on the diagonal of the image would require a bounding box the size of the image, potentially completely encapsulating other instruments as depicted in Fig. 1.

In this paper, we propose a novel method for instance segmentation and classification of surgical instruments in endoscopic images. We exploit the fact that surgical instruments differ mainly on small parts of the instruments by extracting instances and then classifying them independently. Instruments are initially separated into instances and then independently classified. Our method’s architecture is based on an encoder–decoder network with a shared encoder, two decoders for instance segmentation, and a classifier for instance classification. In comparison with semantic segmentation, our method has distinct advantages. Instance segmentation allows for further processing such as pose estimation [1] or surgical tool tracking [4, 11]. Furthermore, by incorporating the instance segmentation results into the classification task, our method naturally aggregates global visual information from different parts of the image into a instance-wise classifier. This is fundamental to correctly classify elongated objects whose distinctive parts are comparatively small. On the other hand, typical semantic segmentation architectures have difficulties to convey global visual information to all the pixels of their output layers. As we observe experimentally, this behaviour makes them unsuitable for this task.

We evaluate our method on the 2017 endoscopic vision robotic segmentation challenge and see an increased segmentation performance of 18% compared to previous approaches based on either bounding boxes or semantic segmentation.

**Fig. 2:**
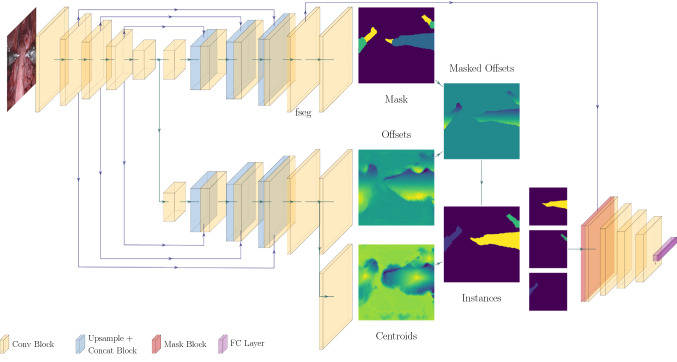
Network architecture. A shared encoder feeds into two decoder heads for instance segmentation and a shallow CNN for type classification

## Methods

The main idea of our method is to perform instance segmentation without bounding boxes and then classify each found instance independently. Formally, given an input image $X \in {\mathbb {R}}^{h\times {}w}$, our goal is to segment the image assigning to each pixel a tuple $(i, c) \in {\mathbb {N}}\times {}{\mathcal {C}}$, where $i\in {\mathbb {N}}$ is an integer indicating the instance number of the instrument occupying that pixel, and $c\in {\mathcal {C}}= \{0, \ldots , C\}$ describes the type of the instrument. The special value (0, 0) is reserved for background pixels. We proceed in a sequential manner: we first perform pixel-wise instance segmentation of the surgical instruments and then leverage the information contained in the instance masks to classify each instrument independently. Our method, as shown in Fig. 2, is therefore a composition of two components: an *instance segmentation* module that produces *i*, and an *instrument classifier* that uses the instance masks to produce *c*. By doing so, we ensure that the classifier labels each instance by incorporating all relevant image information while ignoring background statistics.

### Instance segmentation

As explained, the nature of surgical images and the typical position of the surgical instruments make bounding boxes unsuitable for instance segmentation. In this domain, instance segmentation should be performed in a pixel-wise manner without bounding boxes. To this end, our instance segmentation component learns and combines three intermediate pixel-wise representations of the input image to produce the final pixel-wise instance segmentation ${\hat{y}}_{\text {ins}}$: an instrument segmentation ${\hat{y}}_\text {s}$, an offset regression ${\hat{y}}_\text {o}$, and a centroid heatmap ${\hat{y}}_\text {c}$.

The instance segmentation ${\hat{y}}_\text {s}$ could be, in principle, a binary mask that labels each pixel as *instrument* or *background*. In practice, however, we choose to produce a richer segmentation of instrument parts. Producing segmentations of instrument parts is not only more useful as a standalone output, but can also help to produce more descriptive feature maps that will be used in the instrument classifier as we explain below. Thus, each pixel of ${\hat{y}}_\text {s}$ is assigned a label in the range $\{0, \ldots , P\}$, which indicates that the pixel belongs to one of the *P* instrument parts, or 0 for background. We train the network to produce accurate segmentations by minimizing both the cross-entropy and the DICE losses of the predictions,

1 $$\begin{aligned} L_\text {s}({\hat{y}}_\text {s}, y_\text {s}) = \text {CE}(y_\text {s}, {\hat{y}}_\text {s}) + \text {DICE}(y_\text {s}, {\hat{y}}_\text {s}), \end{aligned}$$

where $y_\text {s}$ are the manual ground-truth annotations.

The offset regression ${\hat{y}}_\text {o}$ and the centroid heatmap ${\hat{y}}_\text {c}$ follow previous works [10, 16] on instance segmentation. Each pixel ${\mathbf {p}}$ of the offset regression ${\hat{y}}_\text {o}$ is an offset vector that points from ${\mathbf {p}}$ to the centroid of the instance that occupies the pixel ${\mathbf {p}}$. The training minimizes the L1-norm between the predicted and real offsets $y_\text {o}$ ignoring the offsets predicted in background pixels,

2 $$\begin{aligned} L_o({\hat{y}}_\text {o}, y_\text {o})= & {} \dfrac{1}{\sum _{p} \mathbb {1}[y_\text {s}({\mathbf {p}}) \ne 0] } \sum _{{\mathbf {p}}} \mathbb {1}[y_\text {s}({\mathbf {p}}) \ne 0] \nonumber \\&\quad \times \, \Vert {\hat{y}}_\text {o}({\mathbf {p}}) - y_\text {o}({\mathbf {p}}) \Vert _1. \end{aligned}$$

Simultaneously with the offset regression, the network learns to predict the centroid heatmap ${\hat{y}}_\text {c}$ that estimates the locations of the centroids of all visible instruments in the image. Finding the centroids besides the offsets seems redundant, but we have found that it reduces the complexity of assigning pixels to instances, as without centroids the offset predictions would first have to be clustered with an unknown number of instances. The ground-truth $y_\text {c}$ of the centroid heatmap is generated by placing unnormalized Gaussian functions centered on the median of each instance mask and setting $\sigma =20$. Where multiple Gaussian functions overlap, the maximum value is used. The training minimizes the penalty-reduced pixel-wise focal loss between the predicted heatmap and the ground-truth as in [19],

3 $$\begin{aligned}&L_\text {c}({\hat{y}}_\text {c}, y_\text {c}) \nonumber \\&\quad = -\frac{1}{N} \sum _{{\mathbf {p}}} {\left\{ \begin{array}{ll} (1-{\hat{y}}_\text {c}({\mathbf {p}}))^{\alpha }log({\hat{y}}_\text {c}({\mathbf {p}})) &{}\quad \text {if } y_\text {c}({\mathbf {p}}) = 1, \\ (1-y_\text {c}({\mathbf {p}}))^{\beta }({\hat{y}}_\text {c}({\mathbf {p}}))^{\alpha }log(1-{\hat{y}}_\text {c}({\mathbf {p}})) &{}\quad \text {otherwise,} \end{array}\right. }\nonumber \\ \end{aligned}$$

where $\alpha$ and $\beta$ are hyperparameters of the loss.

Finally, combining ${\hat{y}}_\text {s}$, ${\hat{y}}_\text {o}$ and ${\hat{y}}_\text {c}$ we obtain the desired instance segmentation ${\hat{y}}_{\text {ins}}$. During inference, we first extract a discrete set of centroids ${\mathcal {S}} = \{{\mathbf {s}}_1, \ldots , {\mathbf {s}}_S\}$ from the centroid heatmap ${\hat{y}}_\text {c}$ by performing non-maxima suppression following the method of [19]. We limit the maximum number of extracted centroids to 4, as a maximum of 3 instruments and an additional ultrasound probe can be visible at any given time. The instance *i* of each pixel ${\mathbf {p}}$ is the index of the closest centroid in ${\mathcal {S}}$ according to the predicted offset,

4 $$\begin{aligned} {\hat{y}}_{\text {ins}}({\mathbf {p}}) = {\left\{ \begin{array}{ll} \mathop {\hbox {arg min}}\nolimits _{i} \Vert {\mathbf {p}}- {\hat{y}}_\text {o}({\mathbf {p}}) - {\mathbf {s}}_i \Vert _2 &{}\quad \text {if } {\hat{y}}_\text {s}({\mathbf {p}}) \ne 0, \\ 0 &{}\quad \text {otherwise}. \end{array}\right. }\nonumber \\ \end{aligned}$$

*Network architecture* To produce the three intermediate outputs, we use an autoencoder architecture with skip connections as depicted in Fig. 2. A single encoder processes the input image and two independent decoder heads produce the instrument segmentation ${\hat{y}}_\text {s}$ and the centroid-related predictions (${\hat{y}}_\text {o}$, ${\hat{y}}_\text {c}$). For the encoder, we choose the EfficientNet-B0 architecture [15] due to its high-performance and lightweight nature. We use long skip connections from the encoder to the decoders for the last three encoder blocks. Our decoders are lightweight, with an upsampling operation followed by two conv-norm-relu blocks. Due to the GPU memory limitations, we use group normalization [17] instead of batch normalization, which has shown to work better for small batch sizes. The final convolutional block consists of three conv-norm-relu layers.

### Instrument classification

This component consists of a classifier that predicts the type of each instrument visible in the image. To this end, we leverage the feature maps and the instance segmentation masks produced in the previous component. As shown in Fig. 2, the classifier receives the last feature map $f_{\text {seg}}$ of the ${\hat{y}}_\text {s}$ decoder as input. Since these features are used to segment instrument parts, we expect them to be descriptive enough for the task of instrument classification. However, in order to classify each instrument *i* independently, we mask the feature map $f_{\text {seg}}$ with the predicted instance mask $m_i({\mathbf {p}})= \mathbb {1}[{\hat{y}}_{\text {ins}}({\mathbf {p}}) = i]$. This operation hides irrelevant background information and visual information corresponding to other instruments and keeps only the features of the instrument instance *i*. Formally, the input to the classifier is the collection of pixel-wise products $f_{\text {seg}}\cdot m_i$ for all different instances *i* contained in ${\hat{y}}_{\text {ins}}$. Note that, by abuse of notation, the pixel-wise product is applied over all channels of $f_{\text {seg}}$. We pass the masked features through a shallow classification CNN. The classifier outputs the probability distribution ${\hat{y}}_\text {t}$ over the set of instrument types. The training minimizes the weighted cross-entropy loss between the predicted probabilities and the real class,

5 $$\begin{aligned} L_{\text {t}}({\hat{y}}_\text {t}, y_\text {t}) = -\sum _c w_c \cdot y_\text {t}(c) \cdot \log {\hat{y}}_\text {t}(c), \end{aligned}$$

where the weights $w_c$ are the inverse instrument type class occurrence statistics and the ground-truth $y_\text {t}$ is the one-hot encoding of the true class.

*Network architecture* The classification network uses four conv-norm-relu layers followed by a max-pooling with a stride of 2. Finally, a global max-pooling layer summarizes the spatial dimensions and a fully connected layer produces the final prediction. The input feature map contains 32 channels.

### Training

Training minimizes the sum of the joint loss function end-to-end,

6 $$\begin{aligned} {\mathcal {L}} = L_\text {t} + L_\text {o} + L_\text {c} + \gamma L_\text {s}, \end{aligned}$$

where $\gamma$ is set to a large value to enforce good segmentation masks. Given that during training, especially in early epochs, the predicted segmentation ${\hat{y}}_{\text {ins}}$ contains lots of inaccuracies, we use the ground-truth instance segmentation $y_{\text {ins}}$ as input to the classifier instead of the predicted ${\hat{y}}_{\text {ins}}$ to prevent it from learning from invalid features. We additionally perturb the input feature maps $f_{\text {seg}}$ with dropout in order to prevent overfitting.

**Table 1:** Results on the fourfold CV for instrument parts and types

Method	Parts mIoU	Types mIoU	Types mIoU test
TernausNet [13]	$0.655 \pm 0.172$	$0.338 \pm 0.192$	–
MF-TAPNet [9]	$0.679 \pm 0.165$	$0.362 \pm 0.228$	–
ISINet [5]	–	0.536*	–
ISINet-temporal [5]	–	0.556*	–
Ours	$0.646 \pm 0.036$	$\mathbf{0}.657 \pm 0.075$	$0.720 \pm 0.070$

We include the results on the test sequences 9 and 10 as well

*No standard deviation provided by authors

Bold indicates the best performing method

The clasper visibility is fundamental to correctly identify the type of an instrument, and we found that training the classifier when claspers are not visible leads to degraded performance. Therefore, as an additional measure during training, we filter out from the classification task any instrument whose clasper is not visible. Images that do not contain any visible clasper at all are entirely discarded from training.

## Experiments

We evaluate the performance of our method on the 2017 Endoscopic Vision robotic segmentation challenge [2]. The dataset consists of eight 225 frame video sequences recorded using the Intuitive Surgical da Vinci Xi system. In addition, two 300 frame sequences are also provided, which we denote as sequences 9 and 10. Annotations are provided on a pixel level for binary, parts and type classes. In total, there are 7 different instrument types. The output of the final fully connected layer of the classification network is set to reflect these 7 instruments. The dataset contains annotations for 4 different instrument parts *P*; shaft, wrist, clasper and other. We set the number of channels $f_{seg}$ to 32.

In line with existing methods, we follow the evaluation procedure from the challenge [2], where the mean intersection over union (mIoU) is computed only if an instrument is present in the ground-truth. To give a complete overview of the performance, we further compute the mean average-precision (mAP) score using the object masks (IoU = 0.50:0.95).

**Fig. 3:**
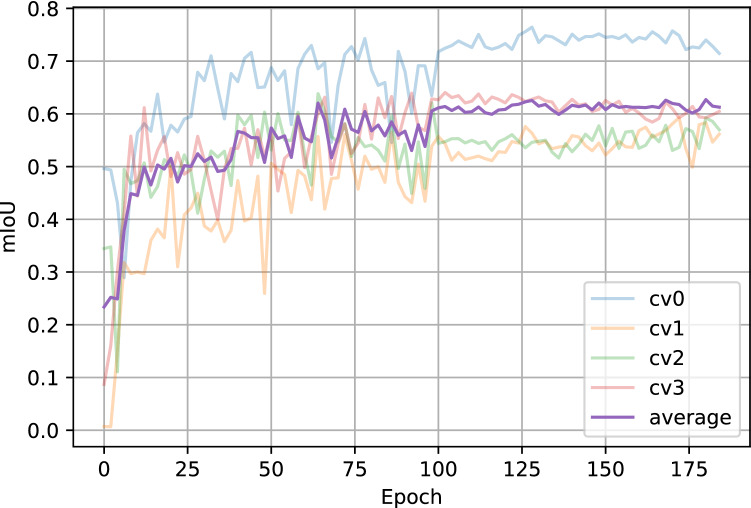
Validation curves for the fourfold cross-validation. We plot the average mIoU score as an approximate lower bound for our method

We perform two families of experiments; (1) a comparison of our method to prior-work using a fourfold cross-validation, and (2) an ablation study to analyse the impact of model design choices on the performance. For the fourfold cross-validation, we follow the data split proposed by [13]. We expand this evaluation by further testing every split on sequences 9 and 10 to provide a less biased estimation of the performance. For the ablation study, we use the single split testing procedure proposed by [8], where sequences 1, 2, 3, 5, 6, 8 are used for training, 4 and 7 for validation and 9, 10 for testing. We use a different split in this experiment to incorporate all previous work which has used datasets 9 and 10 for testing

We train all methods using the AdamW optimizer [12] with a base learning rate of $10^{-3}$ and a batch size of 24 on 6 GPUs. The learning rate is reduced to $10^{-4}$ after 100 epochs. Our encoder is pretrained on ImageNet. To initialize the region masks, we pretrain the ${\hat{y}}_\text {s}$ decoder and the encoder for 20 epochs before optimizing the joint loss. Images are resized to $512 \times 640$ and randomly cropped to $384 \times 480$ for training. Standard augmentation procedures such as translation, rotation and photometric distortions are applied. Methods that were retrained by the authors are used, when possible, in the same training environment, with the same training procedure, optimizer and image augmentation methods. Hyperparameters of these methods were not extensively tuned and, as such, their performance may be slightly understated. For our method, we set the focal loss hyperparameters $\alpha =2$ and $\beta =4$, and the segmentation loss weight $\gamma =10$. We use all images and instances during evaluation, irrespective of clasper visibility.

### Performance comparison

We compare our results with previous works in Table 1. We obtain a mIoU of 0.657, an improvement of at least 18% over the previous state-of-the-art. We note that the validation score suffers from a large variance throughout the training, and picking the best performing model on the leave-out split overstates the performance of our method. We consider the performance of the best model to be an upper bound of the real performance. Figure 3 plots the evolution of the average mIoU across over all 4 splits. To find a lower bound of the real performance, we pick the minimum value reached by the averaged mIoU after the first 125 epochs. This leads to an mIoU of 0.61, still a performance improvement of 11% over the state-of the-art. Furthermore, we state a mAP of $0.499 \pm 0.068$ on the validation and $0.481 \pm 0.099$ on the test set. Using the segmentation head output $y_s$, we further observe a parts mIoU of 0.646, similar to that of previous work. We state a run-time performance of 15 FPS on a NVIDIA 2080 Ti GPU without any specific optimization when three instruments are present.

**Table 2:** Ablation study results (Types mIoU) on sequences 9 and 10

Method	Sequence 9	Sequence 10	Mean
AP-MTL [8]	0.350	0.795	0.573
TernausNet-16* [13]	0.411	0.782	0.596
Mask-RCNN-R50-FPN** [6]	0.327	0.773	0.550
Semantic type segmentation	0.474	0.784	0.629
Single decoder	0.528	0.719	0.623
Binary segmentation	0.612	**0.852**	**0.732**
Proposed method	**0.616**	0.847	0.731

*Open source model used in same training environment

**Implementation from [18]

Bold indicates the best performing method

### Ablation study

In this section, we analyse the impact of a number of design decisions in our method. Specifically, we evaluate the performance of our method with different simplifications:

- *Semantic type segmentation* We remove our instrument classification component. Instead, we add a decoder to our autoencoder architecture to predict ${\hat{y}}_\text {t}$ in a pixel-wise manner as a typical segmentation task. This experiment provides a baseline performance and illustrates the gains yielded by our two-phase mask-then-classify approach.
- *Single decoder* We analyse the effects of using a single decoder instead of separated decoders for instance segmentation. We double the number of channels in the decoder to have comparable computational complexity.
- *Binary segmentation* Instead of finding instrument parts in ${\hat{y}}_\text {s}$, we use binary segmentation masks to distinguish *instrument* from *background*.

**Table 3:** Performance of our method with and without instrument priors

Method	Sequence 9 mIoU	Sequence 10 mIoU	Mean mIoU	mAP
Ours	0.616	0.847	0.731	0.548
Ours with prior	**0.766**	**0.919**	**0.842**	**0.662**

Bold indicates the best performing method

**Fig. 4:**
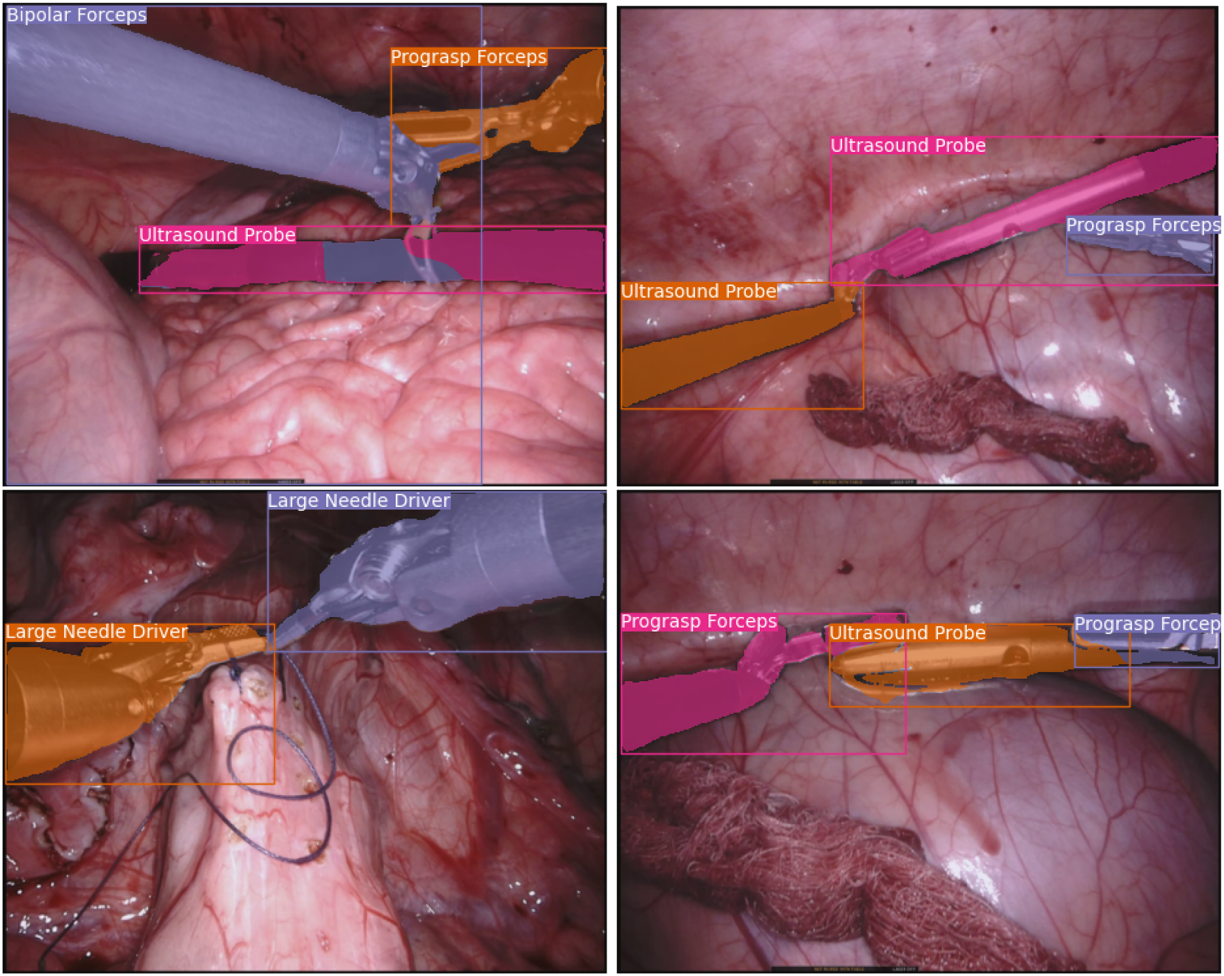
Example of difficult cases with multiple overlaying objects. Typical failure cases can be seen on the top left, where the mask of the bipolar forceps is leaking into the ultrasound probe. On the top right, we see the same issue, with the clasper of the prograsp forcep being included in the ultrasound probe object, resulting in a wrong classification. On the bottom-left, two instruments of the same class overlap with good performance. On the bottom right, two instruments overlap an ultrasound probe showing minor errors in the segmentation mask

**Fig. 5:**
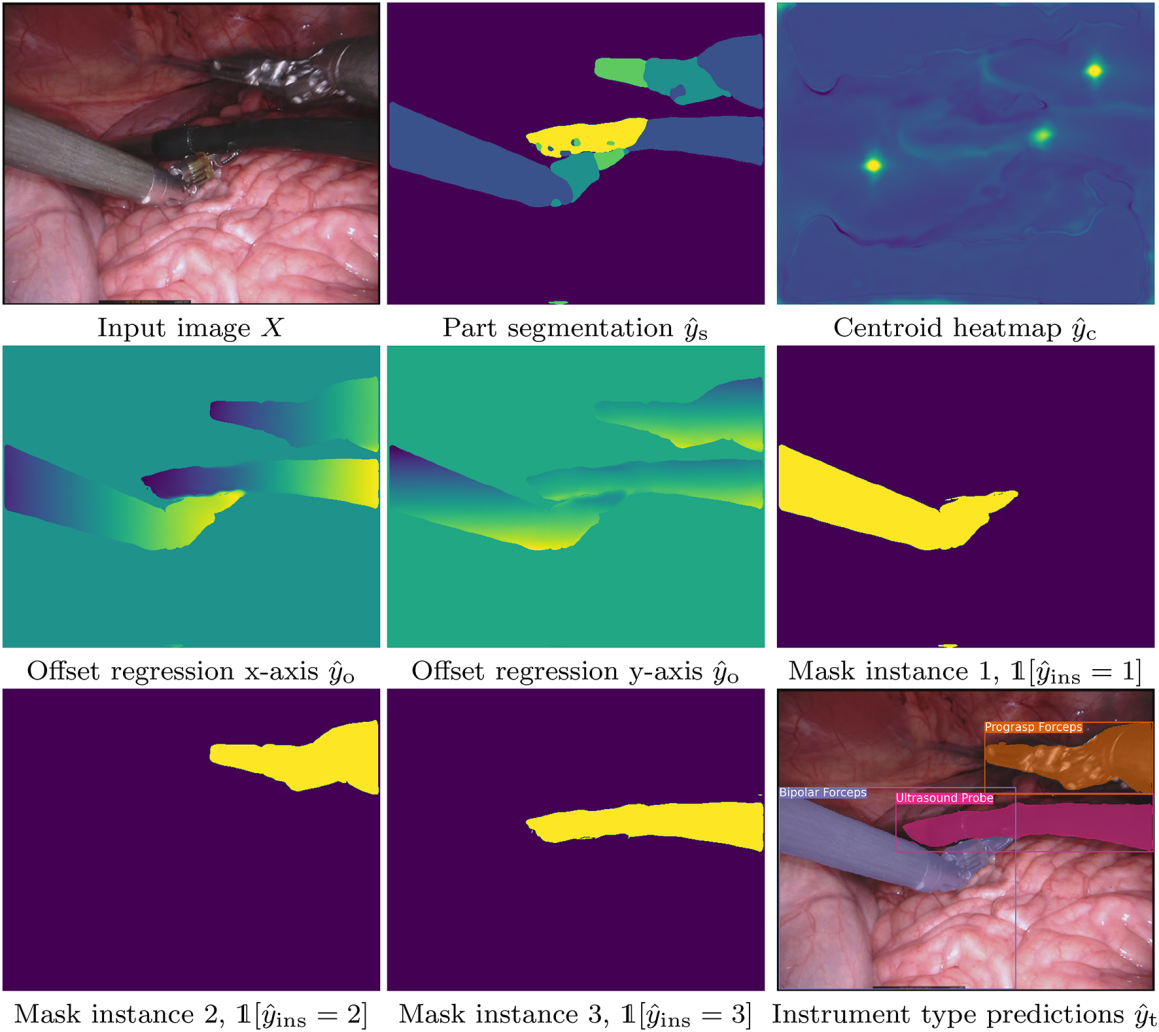
Example of the instance segmentation pipeline. Colour map part segmentation: background: purple, shaft: blue, wrist: cyan, clasper: green, other: yellow. Colour map centroid heatmap: brighter colours indicate higher values. Colour map offset: dark colours indicate lower values, brighter colour higher values

**Fig. 6:**
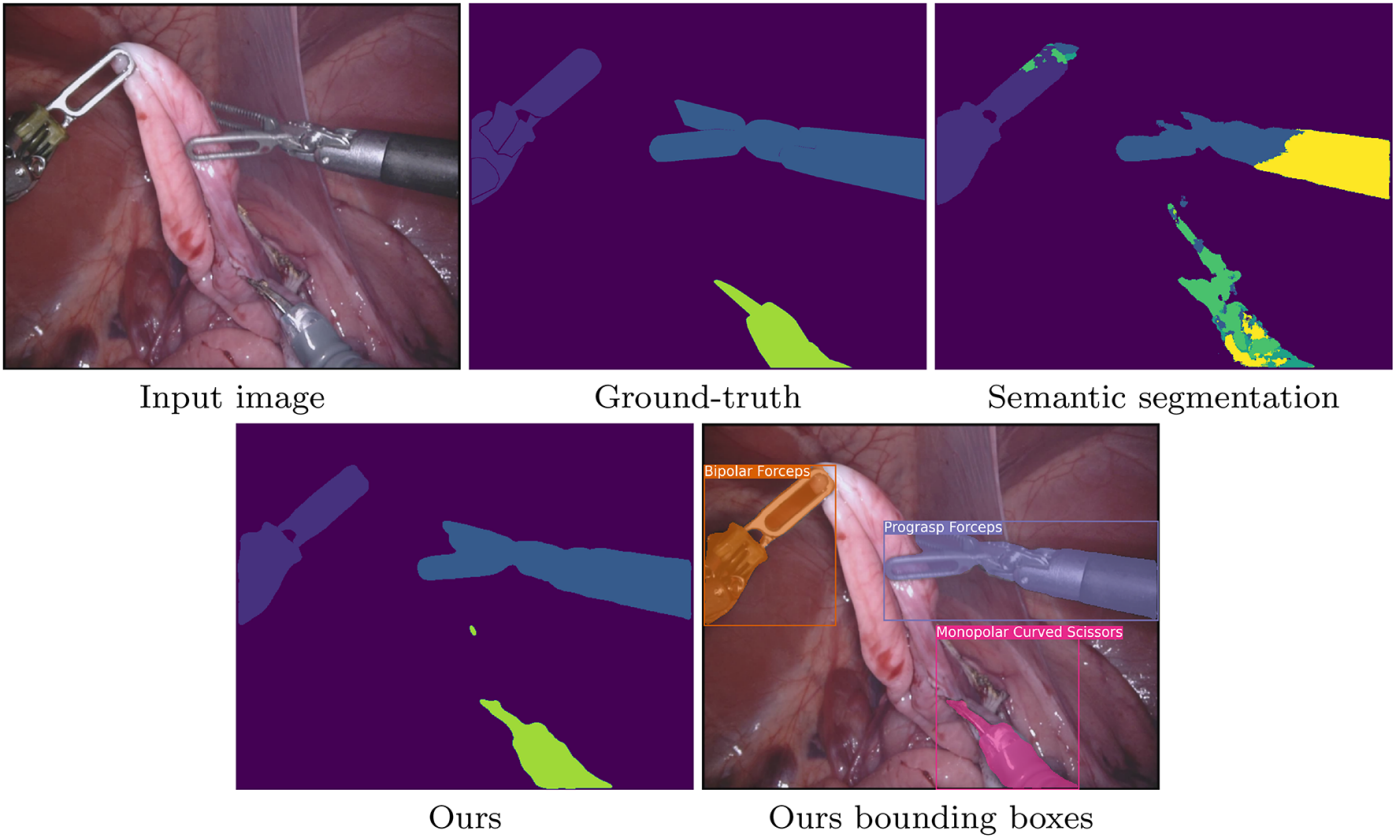
Comparison between standard semantic segmentation and our mask-then-classify method for instrument type classification. The semantic segmentation is very noisy and unable to classify the bottom scissor tool and the shaft of the prograsp forceps. From top-left to bottom right: Input image, ground truth instrument type segmentation, semantic segmentation prediction, proposed method prediction, bounding box visualization

We show the results of the ablation study of our proposed method in Table 2. We also compare these results to three prior methods: the bounding-box-based instance segmentation methods AP-MTL [8] and Mask-RCNN [6], and the method TernausNet-16 from [13], which uses semantic segmentation to find instrument types. The three methods are trained and tested on the same data split as ours.

All our ablated methods outperform prior work, which suggests that our two-component architecture is better suited for this problem. Our complete method also outperforms the semantic type segmentation alternative by over 10% points, whilst performing the more difficult task of instance segmentation. We observe a similar improvement with respect to the model with a single decoder, which justifies our decision to use two decoder heads. Rather surprisingly, the binary segmentation alternative reaches better performance than our complete model with instrument part segmentation. However, this improvement is marginal with a difference of just 0.1% points that could be attributed to statistical noise. On top of that, the instrument part segmentation is interesting by itself as a standalone output and can be useful in practice for tasks such as instrument pose estimation. Therefore, we chose to keep it in the final model.

### Including priors from kinematics

In most robotic applications, the types of the instruments connected to the robot are known at any given point. When this prior knowledge is available, we could, in principle, incorporate it as an additional input to our model to boost the performance. To do so, we build a binary vector indicating the instruments attached to the robot and concatenate it to the max-pooled feature vector of the instrument classification CNN. To account for this additional input, we need to include a second fully connected layer in the network. In Table 3, we compare the results with our base model and see an increase in segmentation performance of over 15% and 20% in mAP.

### Qualitative results

In this section, we highlight some qualitative results of our method. For visualization purposes, we make use of bounding boxes which were computed with the instance maps and type classification. Figure 4 displays some difficult cases for our method. While the model is capable of separating instances in most cases, we still observe that certain overlapping parts of the instruments are attributed to wrong instances. Figure 5 shows intermediate and final results of our pipeline for an input image. Our method is in this case able to separate all three instances and perform a correct classification. The bounding box of the bipolar forceps is skewed due to a small region incorrectly segmented as part of an instrument in the bottom part of the image. In Fig. 6, we compare our method to standard semantic segmentation and show a typical failure case of the latter. The prograsp forceps on the right are segmented into two instruments, where the clasper is correctly identified and the shaft incorrectly identified. The semantic segmentation method is therefore capable of distinguishing instrument claspers, but does not propagate sufficient global context to the shaft to classify it correctly. Our method, on the contrary, shows exceptional performance in this example.

## Discussion and conclusion

In this paper, we propose a novel method for instrument multi-instance segmentation in the scope of minimally invasive robotic surgery. Our method bases on a segment first, classify last approach which, compared to common methods such as MaskRCNN, do not require a bounding box around an object. We compare our method to previous approaches and show that in a fourfold cross-validated experiment our method improves the state-of-the-art by over 18%. Furthermore, we perform an ablation study highlighting the importance of design decisions. Our proposed method here increases the score by over 10% to the baseline segmentation method using the same encoder–decoder structure.

While the results of the experiments show a clear improvement over previous methods, we wish to add some reasoning to this improvement and discuss some key limitation of the method. The method exploits the fact that the instrument type is in most cases classified solely due to the clasper. This is in strong contrast to natural images, where for instance a car differs on most pixels compared to a pedestrian. Together with the large variance in size of the surgical instruments, semantic segmentation methods struggle to sufficiently provide context over large spatial regions. Applications which fit this criteria will most likely benefit from the approach shown in this paper. The major limitation of the method is, however, that all three parts (mask, instance, classification) must work in order to get good results. If for example the instances cannot be separated, the classification network will at best provide a correct label for one instrument and at worst fail completely. Analysing the results, we still see a large margin of improvement which is possible compared to parts and binary segmentation methods. While we have closed the performance gap partially, the method still fails at classifying in many cases. This becomes apparent when adding the instrument presence prior where the performance increases by 15%. We believe that this difference could be reduced by increasing the size of the dataset, as the eight sequences are strongly correlated. In the future, we wish to extend our method to panoptic segmentation where uncountable objects, such as tissue, blood or surgical scaffolds are also incorporated in the prediction output. Furthermore, the method currently does not leverage temporal information which could be fused to improve the performance and consistency. A possible approach could be to track the centroids together with the predictions over time.

## Supplementary Information

Below is the link to the electronic supplementary material.

Supplementary material 1 (mp4 33700 KB)

Supplementary material 2 (mp4 37645 KB)

Supplementary material 3 (mp4 33086 KB)

## Data Availability

All data used are publicly available.

## Code availability

Code is available at: https://github.com/aimi-lab/mask-then-classify.
